# miR‐221/222 Facilitate Pituitary Adenoma Progression Via PHACTR4 Downregulation

**DOI:** 10.1155/humu/8584408

**Published:** 2026-02-05

**Authors:** Wenjing Dong, Shiju Yan, Lingyun Song, Wei Wang, Di Sun, Ping Pang, Guoqing Yang, Weijun Gu

**Affiliations:** ^1^ Department of Endocrinology, Hainan Hospital of Chinese PLA General Hospital, Sanya, Hainan, China, 301hospital.com.cn; ^2^ Department of Endocrinology, First Medical Center of Chinese PLA General Hospital, Beijing, China, 301hospital.com.cn; ^3^ Department of Orthopedics, Hainan Hospital of Chinese PLA General Hospital, Sanya, Hainan, China, 301hospital.com.cn

**Keywords:** microRNA-221/222, migration, PHACTR4, pituitary adenoma, proliferation

## Abstract

Most of pituitary adenomas are biologically benign, but some grow local‐invasively and can invade important adjacent tissues, resulting in clinical symptoms such as hormone secretion disorders and visual field defects. MicroRNA‐221/222 (miR‐221/222) is tandemly encoded on the X chromosome in humans, mice and rats, and is highly conserved in vertebrates with the same seed sequence. To date, miR‐221/222 has been reported as either a tumor suppressor or a tumor promoter in different tumors, however, its role in pituitary tumors has not been elucidated. Our study aimed to investigate the effect and mechanism of miRNA‐221/222 in pituitary tumor cells. Results of real‐time quantitative PCR showed that the expression level of miRNA‐221/222 in plasma exosomes from patients with pituitary tumor was significantly higher than that from healthy people. Results of cell function experiments indicated that miRNA‐221/222 significantly promoted cell proliferation and migration, inhibited apoptosis and significantly inhibited the expression of Cleaved‐Caspase3 and E‐cadherin, while promoted the expression level of N‐cadherin. With transcriptome sequencing and comprehensive bioinformatics analysis, PHACTR4 was identified as the potential target gene of miRNA‐221/222 in regulating biological functions of pituitary adenoma cells. Dual luciferase reporter assay confirmed that PHACTR4 was the direct target gene of miRNA‐221/222 and overexpression of PHACTR4 gene reversed the regulatory effects of miRNA‐221/222. In vivo experiment of subcutaneous tumor formation in nude mice verified that miRNA‐221/222 promoted tumor growth by targeting PHACTR4. In conclusion, miRNA‐221/222 played the role of proto‐oncogene in the occurrence and development of pituitary tumors by targeting PHACTR4, which provided a new target for the diagnosis and molecular treatment of pituitary adenomas.

## 1. Introduction

Pituitary adenomas (PAs) are benign tumors originating from parenchymal cells of the anterior pituitary gland, ranking for the third most common intracranial tumors [1, 2]. A meta‐analysis of epidemiological studies of PA showed that overall prevalence of PAs was about 16.7% (14.4% in autopsy studies and 22.5% in radiology studies) [3]. Most of PAs are slow‐growing and histologically benign tumors without migration and invasion. However, some show local invasive growth, invading sellar floor, optic chiasm, and other important adjacent tissues, causing hormone secretion disorders, visual defects, and other clinical symptoms, showing resistance to conventional treatment and easy relapse after surgery. Transsphenoidal endoscopic resection is first‐line treatment of PA. However, because of the complex adjacent tissue structure, complete surgical resection is challenging in some cases. Some studies have pointed out that 10‐year recurrence rate of PA was about 10% [4]. However, study from Spain found that at least 50% of patients with surgically removed nonfunctioning PA suffered postoperative recurrence [5]. Therefore, finding potential diagnostic markers and therapeutic targets for PA is an urgent problem to be solved in clinical work.

microRNAs (miRNAs) are a class of endogenous noncoding RNAs with 19‐23 nucleotides in length, playing an important role in post‐transcriptional regulation and affecting cell development, differentiation, proliferation, apoptosis, and the pathogenesis and development of tumors [6]. Currently, more than 1000 human miRNAs are known to regulate over 50% of the protein‐coding genes [7]. It has been reported that miRNAs acted as proto‐oncogenes or tumor suppressor genes, and participated in the regulation of tumor epithelial–mesenchymal transition, invasion, and metastasis through different mechanisms and signaling pathways [8–12]. miRNA‐221 and miRNA‐222, homologous miRNAs located on human chromosome Xp11.3, play a significant role in the regulation of various cancers. Studies have found that high level of miRNA‐221/222 was involved in the occurrence and development of thyroid cancer [13]. In breast cancer, miRNA‐221/222 promoted epithelial–mesenchymal transition by regulating Notch3, leading to tumor progression [14]. Moreover, miRNA‐221/222 inhibited cell apoptosis and promoted cell proliferation by inhibiting Caspase‐10, facilitating the occurrence and development of prostate cancer [15]. However, effects of miRNA‐221/222 in PA have not been investigated. Based on this, this study intended to start with the collection of blood samples from patients with clinical PA, screen and verify the potential molecular mechanism of miR‐221/222 in regulating proliferation, apoptosis and migration of pituitary tumor cells by transcriptome sequencing and bioinformatics analysis, so as to provide theoretical basis for new targets for early diagnosis and precise treatment of PA.

## 2. Materials and Methods

### 2.1. Patients

A total of 16 patients (mean age ± SD: 46.8 ± 6.7 years) diagnosed with PA hospitalized in the Department of Endocrinology, Hainan Hospital of Chinese PLA General Hospital from December 2022 to July 2024 were consecutively recruited. Inclusion criteria were as follows: (1) PA was confirmed by brain tomography imaging or pathology and (2) patients with complete medical record. Exclusion criteria were as follows: (1) patients had received chemoradiotherapy; (2) recurrence; (3) with malignant tumors in other parts of body; (4) incomplete medical record; (5) those who refused to sign the informed consent form. Sixteen gender‐ and age‐matched healthy subjects (healthy control group, 48.7 ± 5.2 years) were selected from the same hospital during the same period of time. This study was approved by the Ethics Committee of Hainan Hospital of Chinese PLA General Hospital. Written informed consent was obtained from each subject.

### 2.2. Cell Culture

Rat PA cell lines GH3 and mice PA cell lines GT1‐1 were purchased from Fuheng Biotechnology (Shanghai, China). GH3 cell lines were cultured in EMEM medium (Hyclone, United States) containing 2.5% FBS (Gibco, United States) and 15% HS (Gibco, United States) while GT1‐1 cell lines were cultured in DMEM/F12 medium (Hyclone, United States) containing 8% FBS (Gibco, United States) and 2% HS (Gibco, United States) in an incubator with a constant temperature of 37°C and 5% CO_2_.

### 2.3. Cell Transfection

miR‐221 mimic, miR‐221 inhibitor, miR‐222 mimic, miR‐222 inhibitor, and nonspecific negative control (NC) oligonucleotides were synthesized and purified by Asia‐Vector Biotechnology (Shanghai, China) (Table S1).

When GT1‐1 and GH3 cells reached 80% confluence, miR‐221/222 mimic, miR‐221/222 inhibitor, or nonspecific NC were transfected at a working concentration of 100 nM using Lipofectamine 3000 (Invitrogen) according to the manufacturer’s protocol.

### 2.4. Exosome and RNA Extraction, Reverse Transcription, and RT‐qPCR

#### 2.4.1. Exosome Isolation

Exosome was isolated from plasma from patients using ultracentrifugation (UC). Briefly, plasma was centrifuged (Beckman Coulter, United States) at 3000 ×*g* for 10 min at 4°C to remove cell debris. The pellet was washed in PBS and then centrifuged 16,000 ×*g* for 70 min at 4°C to remove remaining macrovesicles. The sediment was re‐suspended in PBS and centrifuged at 110,000 ×*g* for another 70 min to remove contaminant proteins.

#### 2.4.2. RNA Extraction, Reverse Transcription, and RT‐qPCR

Total RNA was extracted from the exosomes and cells by TRIzol reagent (Thermo Fisher Scientifc, United States). Concentration of the extracted RNAs were determined with NanoDrop 2000 spectrophotometer (Thermo Scientific, United States). miRNA was analyzed using All‐in‐One miRNA First‐Strand cDNA synthesis kit and miRNA qPCR kit (GeneCopoeia, United States). miRNA‐16 was used as endogenous control for miRNA detection: 5 ^′^‐ TAGCAGCACGTAAATATTGGCG‐3 ^′^ (forward). Total RNA was reverse‐transcribed to generate first‐strand cDNA using cDNA reverse transcription kits (Applied Biosystems, United States) according to the manufacturer’s instructions. Real‐time PCR was performed to detect mRNA level of target genes with Power SYBR‐Green PCR Master Mix and 7900 HT Fast Real‐Time PCR system (Applied Biosystems, United States). GAPDH was used for the normalization of mRNA expression.

### 2.5. CCK‐8 Assay

Twenty‐four hours after transfection, GT1‐1 and GH3 cells were harvested and seeded into 96‐well plates (Corning) at a density of 5 × 10^3^/well. After culturing for 0, 24, 48, and 72 h, Cell Counting Kit‐8 (CCK‐8; Sangon Biotech, China) reagent was added to the corresponding wells and incubated for 3 h. The absorbance was measured at a wavelength of 450 nm by a Multiskan microplate reader (Thermo Fisher, United States).

### 2.6. Cell Apoptosis Assay

Cell apoptosis was detected with Annexin V‐FITC Apoptosis Detection kit (BD Biosciences, United States) according to the manufacturer’s protocol. Briefly, 48 h after transfection, 1 × 10^5^ treated cells of each group were collected, washed, and incubated with Annexin V‐FITC and propidium iodide (PI) for 15 min at room temperature of 20°C in the dark. Cells were analyzed using a fluorescence‐activated cell sorting (FACS) flow cytometer (BD Biosciences, United States).

### 2.7. Transwell Assay

Standard 24‐well Transwell chamber with 8.0 *μ*m pore membrane (Corning) was used to perform the Transwell migration assay; 5 × 10^4^ transfected GT1‐1 and GH3 cells were harvested and seeded on top of the Transwell membrane in culture medium without FBS. In the bottom layer of the 24‐well plate, 500 *μ*L of culture medium supplemented with 100 *μ*L of FBS was added. After 24 h of incubation, cells that migrated through the upper layer were fixed using 4% formalin for 20 min and stained with crystal violet for visualization counting and statistical analysis.

### 2.8. Western Blot Analysis

Forty‐eight hours after transfection, chondrocytes were rinsed with PBS and treated with RIPA lysis buffer (Beyotime, China) supplemented with enzyme inhibitor cocktail (Roche, Switzerland) on ice for 30 min. Total protein concentrations were calculated using BCA protein assay kit (Beyotime, China). Twenty‐five micrograms of total protein for each sample was loaded and separated by 10% SDS‐PAGE and transferred to PVDF membranes (Millipore, United States). Membranes were blocked with 5% skim milk and then incubated with primary antibodies against cleaved Caspase3 (1:1000, #4695S, CST), Caspase3 (1:1000, #4370S, CST), E‐cadherin (1:1000, #9252S, CST), and N‐cadherin (1:1000, #4671S, CST), respectively, overnight at 4°C. Membranes were incubated with secondary antibodies for 2 h at room temperature after membranes were rinsed with TBST. Protein bands were visualized using the ECL luminescent reagent (Millipore, United States) and analyzed using ChemiDoc Imaging System (Bio‐Rad, United States) with Quantity One analyzing system.

### 2.9. Transcriptome Sequencing and Selection of Differentially Expressed Genes

GT1‐1 cells were divided into four groups and transfected with miRNA‐221/222 mimic, miRNA‐221 mimic, miRNA‐222 mimic, and NC, respectively. Each group had three replicates. Forty‐eight hours after transfection, transcriptome sequencing was performed by the Shanghai branch of Zhejiang Xingyi Technology. Sequence alignment of Clean Reads to the indicated reference genome was performed using HISAT2 software. For biological duplicates, DESeq2 was used for differential expression genes (DEGs) analysis. For experiments without biological replicates, edgeR was used for analysis. *p* < 0.05, |log2(foldchange)| > 1 was used as the criterion for screening DEGs. Cluster analysis was used to determine the DEGs among different groups. DEGs screened in each comparison group were subjected to Venn or Upset analysis to screen for unique or shared DEGs. Heat maps and volcano maps were generated using the pheatmap and ggplot2R software, respectively.

### 2.10. GO and KEGG Pathway Analysis

Gene Ontology (GO) and Kyoto Encyclopedia of Genes and Genomes (KEGG) pathway analyses were conducted to characterize the biological functions of the DEGs. Based on the annotation results of GO analyses, DEGs obtained were compared with the reference species’ genome. In this way, all function categories enriched by DEGs were identified through Fisher’s test (*p* < 0.05). Meanwhile, with the reference genome as the background, metabolic and signal transduction pathways that were significantly affected were determined as well by analyzing the degree of each pathway enriched. All analyses were performed using the clusterProfiler software.

### 2.11. Identification and Analysis of Target Genes

The obtained DEGs were further combined with TargetScan database to obtain intersection genes. Data set GSE240781 (PMID: 38643763 [16]) were downloaded from Gene Expression Omnibus (GEO, https://www.ncbi.nlm.nih.gov/gds), to analyze and verify the results of transcriptome sequencing.

Based on the sequencing results, literature review was conducted through PubMed to search for the target genes related with miR‐221/222 family, which have been published in other models to identify the key genes.

### 2.12. Construction of Stably Transfected Cell Lines

Recombinant plasmid vector encoding PHACTR4 or NC, overexpressing miR‐221, miR‐222, miR‐221/miR‐222 were constructed and purchased from Asia‐Vector (Shanghai, China). Plasmid overexpressing miR‐221, miR‐222, miR‐221/miR‐222 were transfected into GH3 cells to obtain stably miR‐221, miR‐222, or miR‐221/miR‐222‐overexpressing GH3 cells.

### 2.13. Dual‐Luciferase Reporter Assay

Luciferase reporter plasmid containing wild‐type 3 ^′^‐UTR of PHACTR4 and mutant‐tape 3 ^′^‐UTR of PHACTR4 were constructed and purchased from Asia‐Vector Biotechnology (Shanghai, China). HEK‐293T cells at 70% confluence were co‐transfected with wild‐type or mutant‐type 3 ^′^‐UTR luciferase reporter plasmid with miRNA‐221 mimic, miRNA‐222 mimic, or miRNA NC, respectively, using Lipofectamine 3000 (Invitrogen). Forty‐eight hours after transfection, luciferase activities were measured using dual luciferase reporter assay system (Promega).

### 2.14. Xenograft Experiment in Nude Mice

Twenty nude mice (5 weeks old) were purchased from Laboratory Animal Center of Animal Center of Hangzhou Medical College and divided into NC group, miR‐221 group, miR‐222 group, and miR‐221+222 group, with five mice in each group. Stably‐transfected GH3 cells (1 × 10^6^) were subcutaneously injected into nude mice. Two perpendicular diameters of implants were measured using calipers to calculate tumor size (mm^3^) as follows: volume (mm^3^) = 1/2 × length × width^2^. At Day 28, all mice were sacrificed after anaesthetization. Tumors were collected and expression level of PHACTR4, Cleaved‐Caspase 3, E‐cadherin, and N‐cadherin were determined with Western blot.

### 2.15. Statistical Analysis

SPSS 26.0 and GraphPad Prism 8.3 software were used for statistical analysis. Continuous variables were expressed as mean ± standard deviation. One‐way ANOVA was used for difference analysis in multiple groups, and Student’s *t*‐test test was used for comparison between two groups. *p* < 0.05 was considered statistically significant.

## 3. Results

### 3.1. miRNA‐221/222 Acted as Proto‐Oncogene in the Occurrence and Development of PAs

#### 3.1.1. miRNA‐221/222 Was Up‐Regulated in Patients with PA

Sixteen patients with PA (five prolactinomas, two somatotropinomas, one adrenocorticotropic tumor, and eight nonfunctional tumors) and 16 healthy subjects were enrolled in this study. Results of real‐time fluorescence quantitative PCR showed that expression levels of miRNA‐221 in plasma exosome in patients with PA (8.75 ± 3.75) were significantly higher than those in healthy control group (1.00 ± 0.40) (*p* < 0.0001) and expression levels of miRNA‐222 in plasma exosome in patients with PA (5.58 ± 2.59) were significantly higher than those in healthy control group (1.00 ± 0.31) as well.

#### 3.1.2. miRNA‐221/222 Promoted Cell Proliferation, Migration, Epithelial‐Mesenchymal Transition, and Inhibited Apoptosis of GT1‐1 and GH3 Cells

Cell proliferation was detected by CCK‐8 assay. Results showed that compared with NC mimic, proliferation rates of GT1‐1 cells transfected with miR‐221 mimic, miR‐222 mimic, and miR‐221/222 mimic were significantly increased (*p* < 0.05). Meanwhile, compared with NC inhibitor, proliferation rates of GT1‐1 cells transfected with miR‐221 inhibitor, miR‐222 inhibitor, and miR‐221/222 inhibitor were significantly inhibited (*p* < 0.05) (Figure 1a,b).

Figure 1microRNA‐221/222 regulated proliferation, migration, apoptosis and EMT in PA cells. (a–b) microRNA‐221/222 promoted proliferation in GT1‐1 cells. (c–d) microRNA‐221/222 promoted migration in GT1‐1 cells. (c, e) microRNA‐221/222 inhibited apoptosis in GT1‐1 cells. (f–g) microRNA‐221/222 downregulated Cleaved‐Caspase3 and E‐adherin and upregulated N‐cadherin. ∗ vs. NC mimic *p* < 0.05; ∗∗ vs. NC mimic *p* < 0.01; ∗∗∗ vs. NC mimic *p* < 0.001; #*p* < 0.05; ##*p* < 0.01; ###*p* < 0.001; & vs. NC inhibitor *p* < 0.05; && vs. NC inhibitor *p* < 0.01; &&& vs. NC inhibitor *p* < 0.001; % vs. miR221 inhibitor *p* < 0.05; %%% vs. miR221 inhibitor *p* < 0.001.

**Figure:**
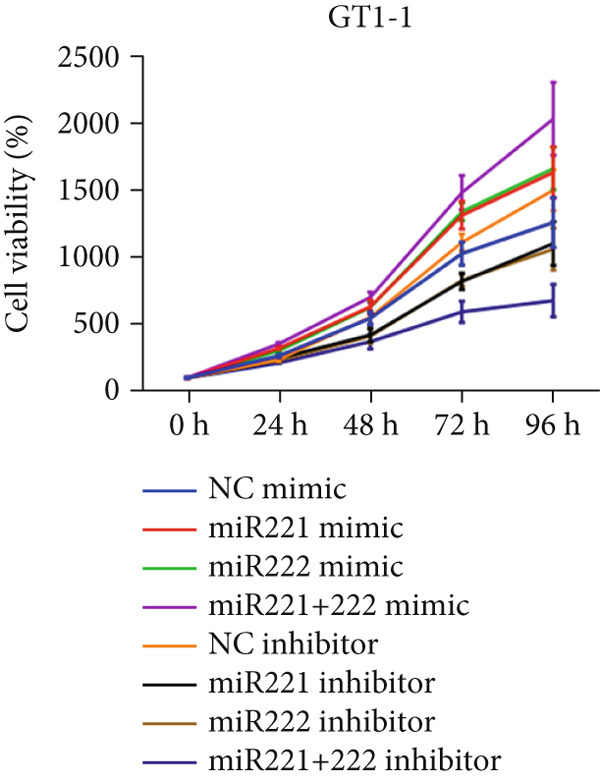
(a)

**Figure:**
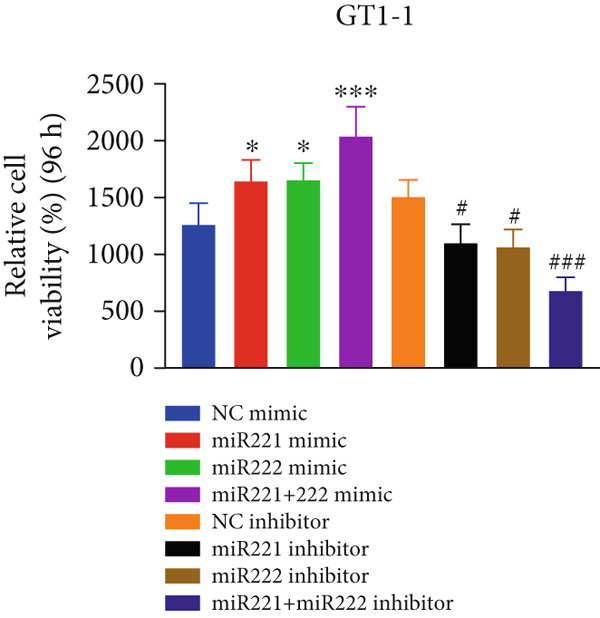
(b)

**Figure:**
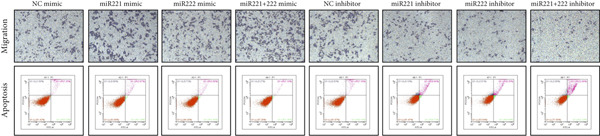
(c)

**Figure:**
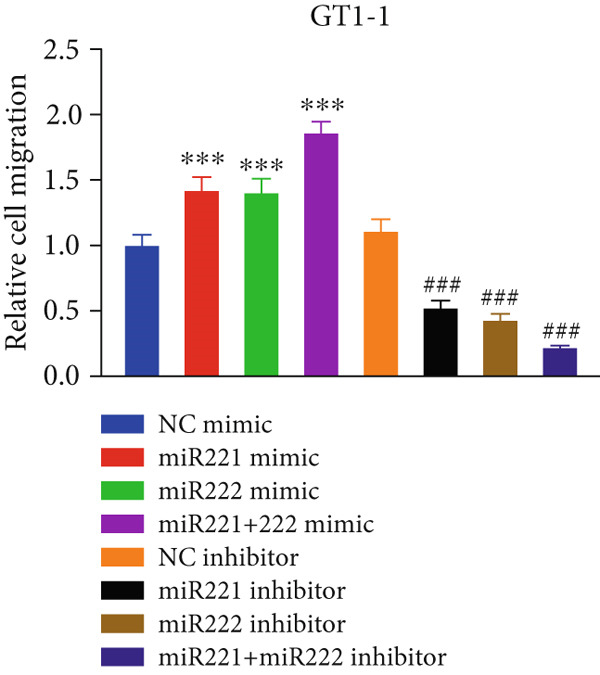
(d)

**Figure:**
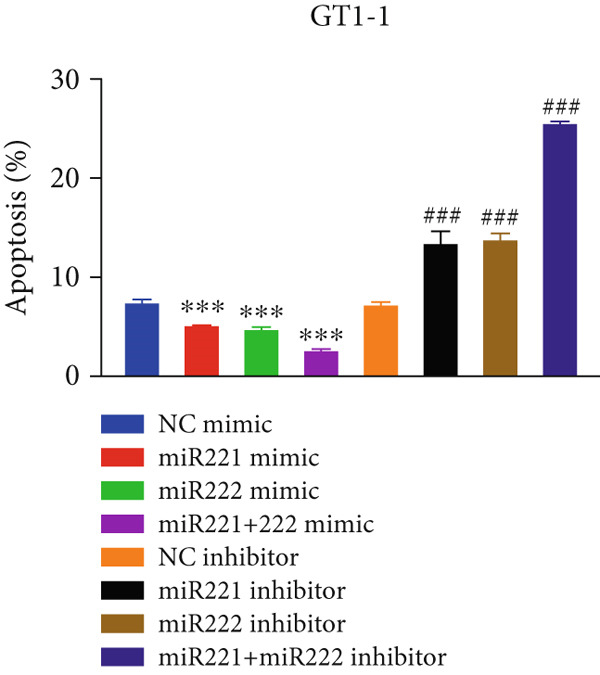
(e)

**Figure:**
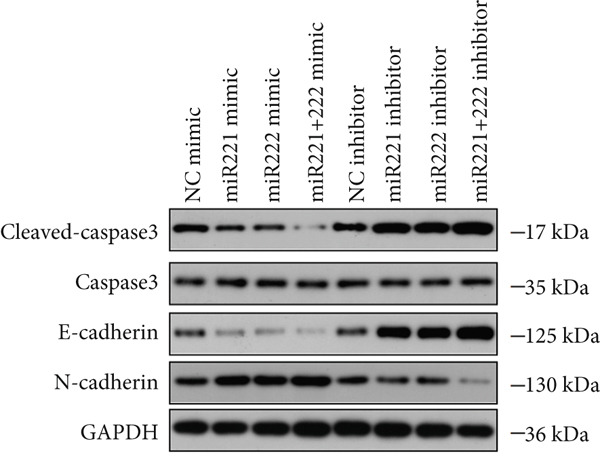
(f)

**Figure:**
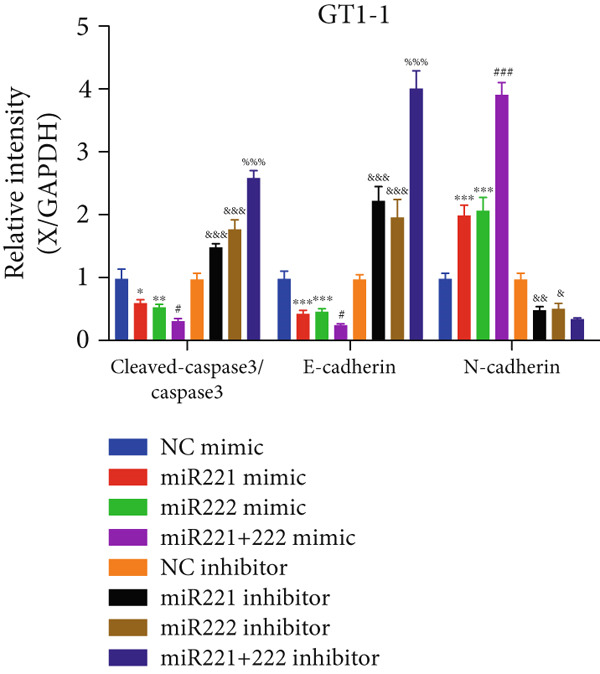
(g)

Similarly, Transwell assay results demonstrated that transfection with miR‐221/222 mimic significantly increased migration of GT1‐1 cells while miR‐221/222 silencing with inhibitor showed the opposite effect (*p* < 0.01) (Figure 1c,d).

Cell apoptosis was detected by flow cytometry after transfection and the results showed that apoptosis of GT1‐1 cells was significantly decreased by miR‐221/222 overexpression (*p* < 0.001), while increased after miR‐221/222 down‐regulation (*p* < 0.001) (Figure 1c,e).

In addition, western blot analysis showed that overexpression of miR‐221/222 inhibited expression levels of Cleaved‐Caspase3 and E‐cadherin, promoted N‐cadherin but cast no effect on Caspase3. While Cleaved‐Caspase3 and E‐cadherin were upregulated and N‐cadherin was downregulated after GT1‐1 cells were transfected with miR‐221/222 inhibitor (*p* < 0.05) (Figure 1f,g).

Results in GH3 cells were shown in Figure S1.

### 3.2. Screening and Validating of Target Genes of miRNA‐221/222 in PA Cells

#### 3.2.1. Identification of DEGs

In this study, nine groups of GT1‐1 cell samples transfected with miRNA‐221 mimic, miRNA‐222 mimic, and miRNA‐221/222 mimic and three groups of NC GT1‐1 cell samples were sent to transcriptome sequencing. After sequencing, a total of 95GB data were obtained. The Q30 base distribution of each sample data was ≥ 92%, the average GC content was 47.98%, and the sequencing error rate was less than 0.01%, without GC bias. *p* < 0.05 and |log2(foldchange)| > 1 were used as the criteria to screen DEGs. The volcano maps of all genes and the expression heatmaps of DEGs were shown in Figure 2a.

Figure 2Screening and validation of target genes of microRNA‐221/222 in PA cells. (a) Expression heatmaps of DEGs of all comparison groups. (b) Venn diagram of intersection differential genes. (c) GO entry bubble map of intersection differentially expressed genes. (d) KEGG enrichment bubble map of intersection differentially expressed genes. (e) Heat map of intersection differential genes. (f) The core genes were imported into the data set GSE240781 to observe the expression of genes in pituitary tumor tissues. Among them, FNIP2, KMT2C, RNF4, AMMECR1, HIPK3, LIFR, PTBP2, PTBP3, KANSL1, TCF12, ARNT, MYO10, WASF2, TIPARP, CRK, HEG1, ZDHHC17, and PHACTR4 were lowly expressed.

**Figure:**
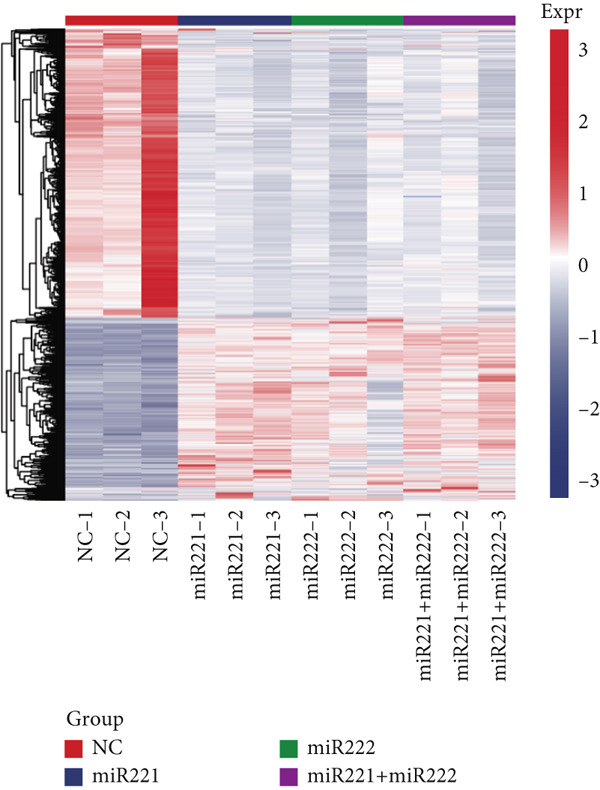
(a)

**Figure:**
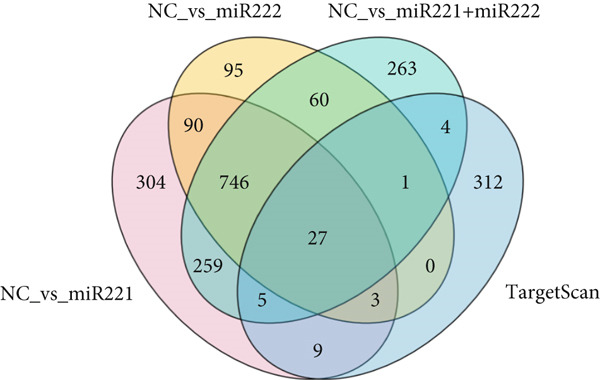
(b)

**Figure:**
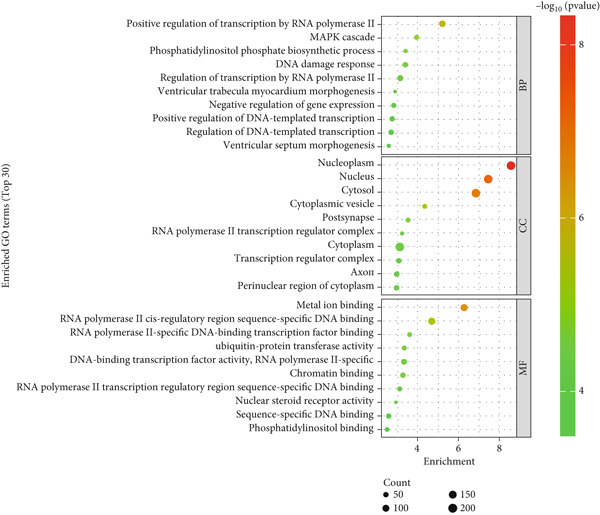
(c)

**Figure:**
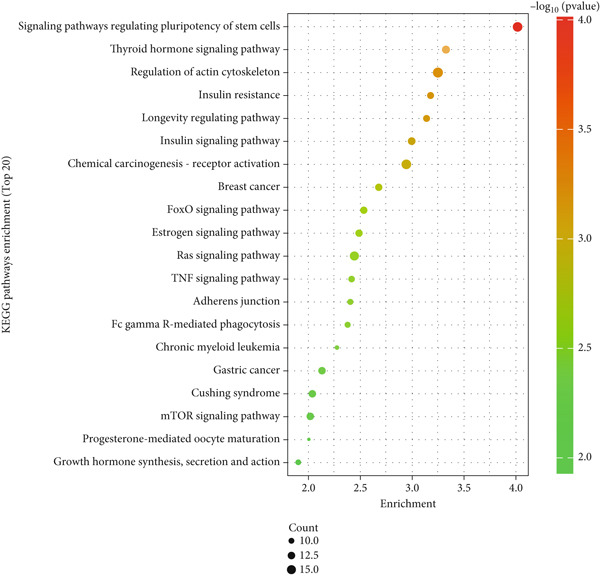
(d)

**Figure:**
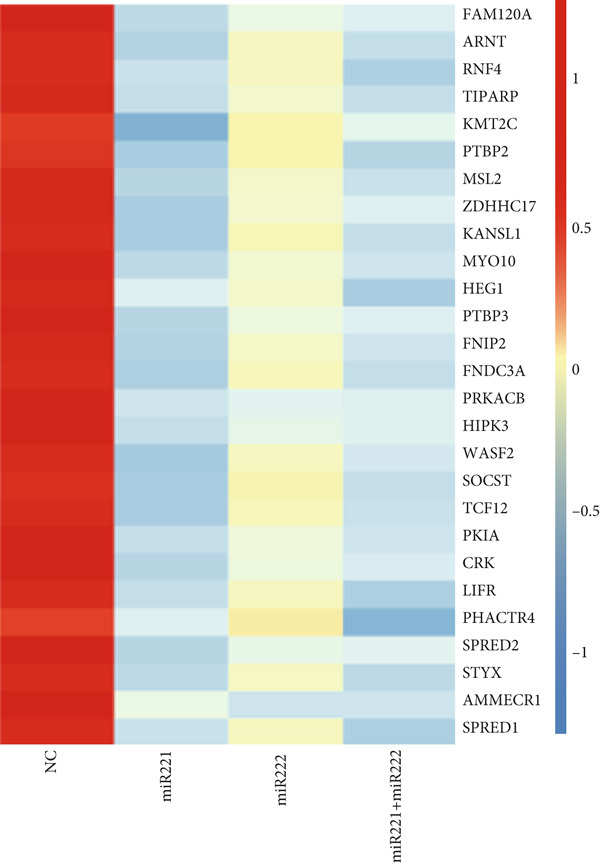
(e)

**Figure:**
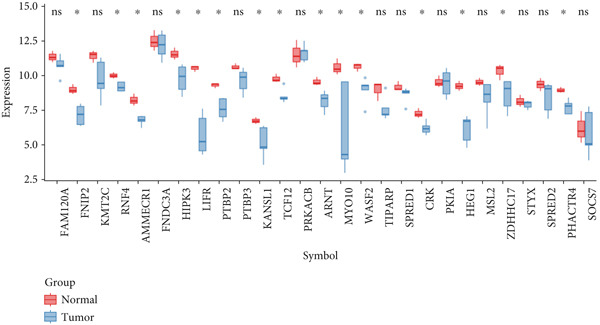
(f)

#### 3.2.2. Identification and Analysis of Target Genes

The DEGs obtained from the analysis of NC vs. miR‐221 mimic, NC vs. miR‐222 mimic, and NC vs. miR‐221/222 mimic were further combined with TargetScan database. Finally, a total of 27 intersection genes were harvested (Figure 2b,c). GO and KEGG analysis were performed on the 27 intersection genes. Biological process items included transcription activation of RNA polymerase II promoter and MAPK cascade activation. Cell component items included nucleoplasm, nuclear and cytoplasmic matrix. Molecular function items included metal ion binding and sequence specific DNA binding of RNA polymerase II cis‐regulatory region. DEGs were significantly enriched in pathways regulating pluripotent stem cell signal transduction pathway and regulation of actin cytoskeleton (Figure 2d,e). Dataset GSE240781 (PMID: 38643763), species of Homo sapiens, annotation platform GPL24676, were utilized to verify the transcriptome sequencing results (Figure 2f). Considering sequencing results and literature review [17, 18], we found that PHACTR4 could be the target gene of miR‐221/222 in PAs.

#### 3.2.3. The Effects of PHACTR4 Overexpression on the Proliferation, Apoptosis, and Migration of PA Cells Transfected With miRNA‐221/222 Mimic

In order to identify PHACTR4 as a potential key gene of miR‐221/222 in regulating the biological behavior of PA cells, PHACTR4 overexpression plasmid was co‐transfected to GT1‐1 and GH3 cells with miR‐221/222 mimic. Cell proliferation, migration, apoptosis, and EMT were measured by CCK‐8, Transwell assay, flow cytometry and Western blot, respectively. Results showed that compared with NC group, miR‐221 mimic, miR‐222 mimic, and miR‐221/222 mimic promoted cell proliferation (Figure 3a,b), migration (Figure 3c,d), and inhibited cell apoptosis (Figure 3c,e), while co‐transfection with PHACTR4 overexpression plasmid attenuated these effects of miR‐221, miR‐222, and miR‐221/222 mimic. In addition, the expression level of PHACTR4, Cleaved‐Caspase3, and E‐cadherin were significantly inhibited while N‐cadherin was significantly increased after miR‐221/222 mimic transfection. Similarly, co‐transfection with PHACTR4 overexpression plasmid awakened these actions of miR‐221, miR‐222, and miR‐221/222 mimic (Figure 3f,g).

Figure 3Effects of PHACTR4 overexpression on the proliferation, apoptosis, and migration of PA cells transfected with microRNA‐221/222mimic. miR‐221/222 promoted cell proliferation (a–b), migration (c–d), and inhibited cell apoptosis (c, e), while co‐transfection with PHACTR4 overexpression plasmid attenuated these effects of miR‐221/222 mimic in GT1‐1 cells. The expression levels of PHACTR4, Cleaved‐Caspase3 and E‐cadherin were significantly inhibited while N‐cadherin was increased after miR‐221/222 mimic transfection and co‐transfection with PHACTR4 overexpression plasmid awakened these actions of miR‐221/222 mimic (f–g). ∗∗ vs. NC Vector *p* < 0.01; ∗∗∗ vs. NC mimic Vector *p* < 0.001; # vs. miR221 mimic Vector *p* <0.05; ## vs. NC + PHACTR4 *p* < 0.01; ### vs. miR221 mimic Vector *p* < 0.001; $$$ vs. NC miR221 mimic + miR222 mimic Vector *p* < 0.001; && vs. miR‐221 mimic + miR‐222 mimic Vector *p* < 0.01; &&& vs. miR‐221 mimic + miR‐222 mimic Vector *p* < 0.001.

**Figure:**
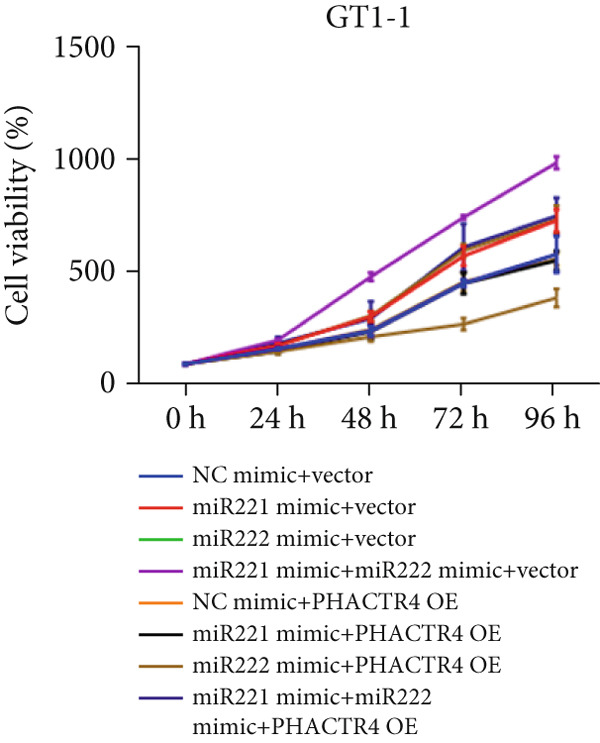
(a)

**Figure:**
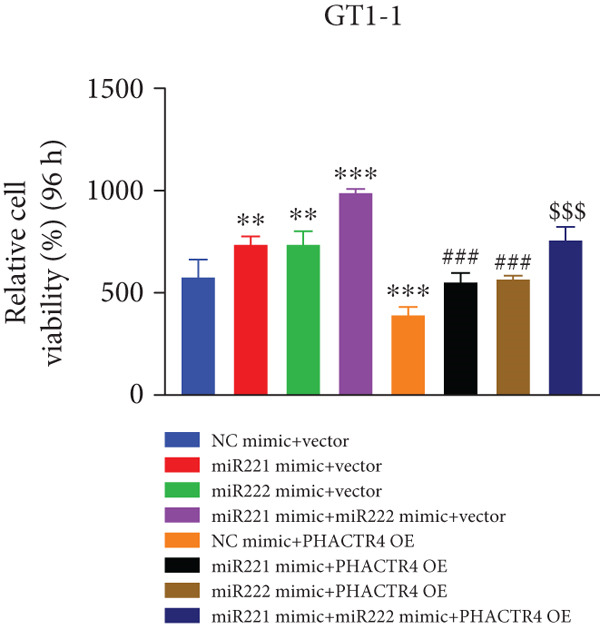
(b)

**Figure:**
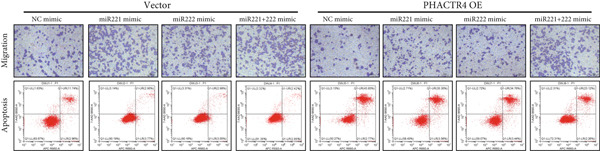
(c)

**Figure:**
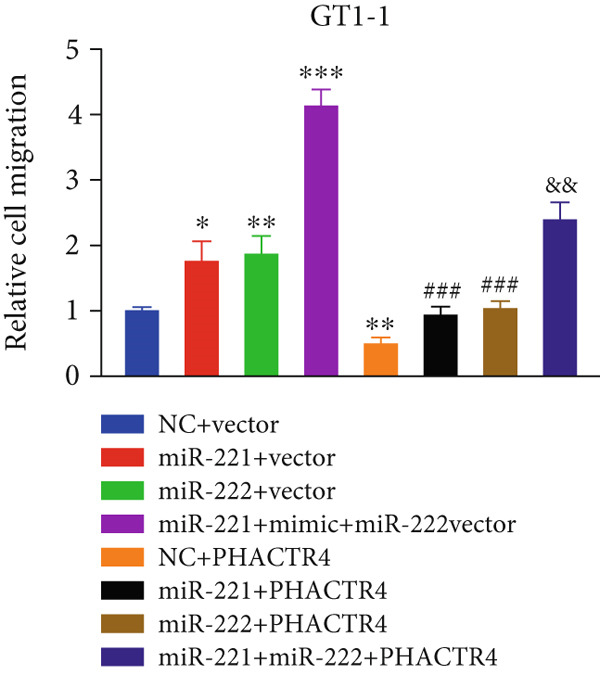
(d)

**Figure:**
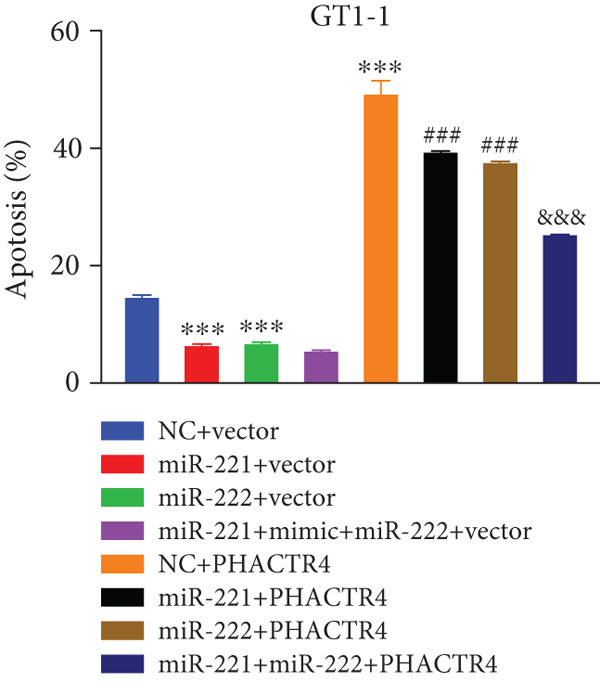
(e)

**Figure:**
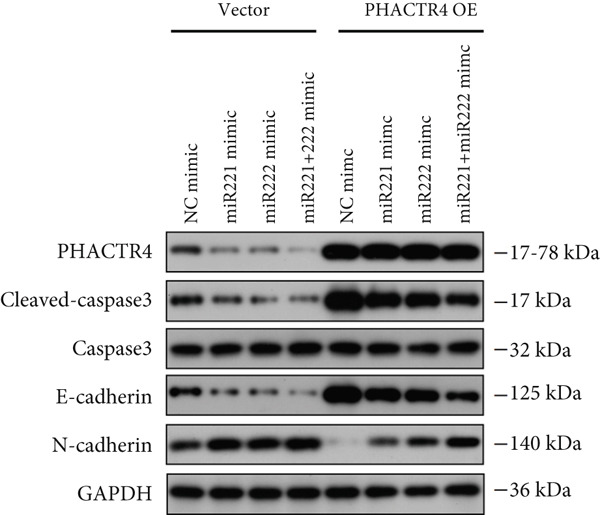
(f)

**Figure:**
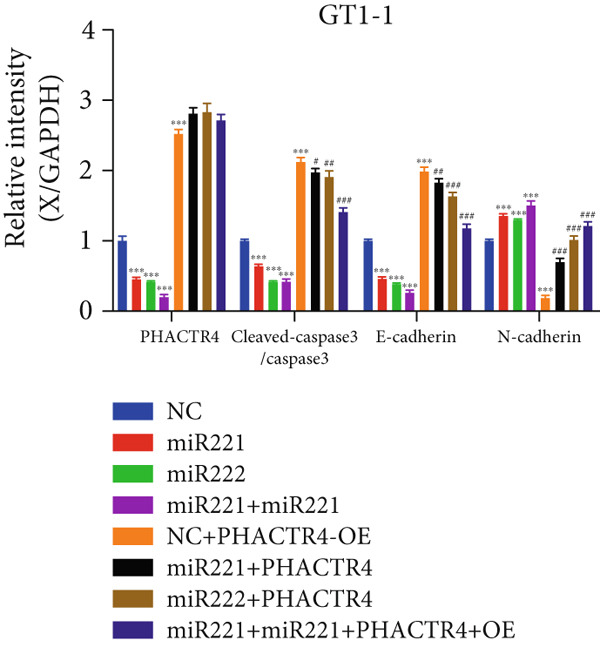
(g)

Results in GH3 cells were shown in Figure S2.

#### 3.2.4. PHACTR4 Was the Direct Target Gene of miR‐221/222

A conserved binding site of miR‐221/222 in PHACTR4 3 ^′^‐UTR was found through TargetScan database (Figure 4a). Western Blot results showed that the expression levels of PHACTR4 in GT1‐1 cells transfected with miR‐221/222 mimic were significantly decreased (*p* < 0.001) and significantly increased in cells transfected with miR‐221/222 inhibitor (*p* < 0.001) (Figure 4c,d). Results in GH3 cells were shown in Figure 4e,f.

Figure 4miR‐221/222 mimic directly targeted PHACTR4 in PA cells. (a) The potential site on PHACTR4 3 ^′^‐UTR for microRNA‐221/222 predicted by Targetscan. (b) Fluorescence intensity of the wild‐type PHACTR4 3 ^′^‐UTR luciferase reporter plasmid was inhibited by transfection of miR‐221/222 mimic while mutation of microRNA‐221/222 binding sites restrained this suppressive effect. (c‐f) microRNA‐221/222 promoted expression of PHACTR4 and microRNA‐221/222 inhibitor inhibited PHACTR4 expression in GT1‐1 cells. (g) Sequences of microRNA‐221/222 in human, mice and rat. ∗∗∗ vs. NC mimic *p* < 0.001; ### vs. NC inhibitor *p* < 0.001.

**Figure:**
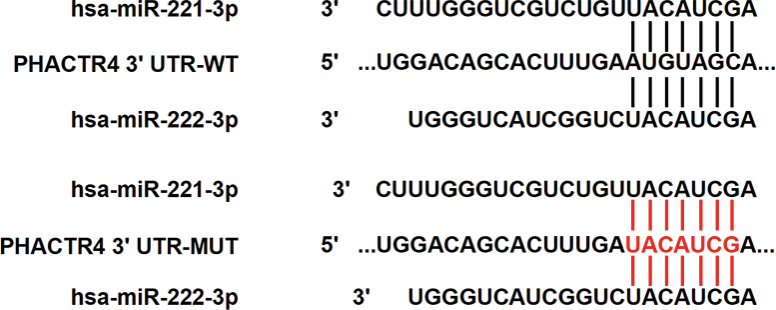
(a)

**Figure:**
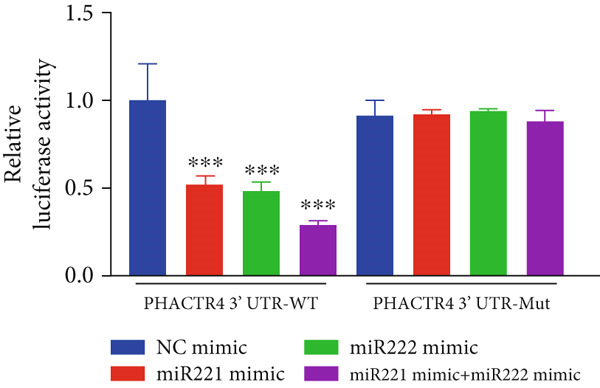
(b)

**Figure:**
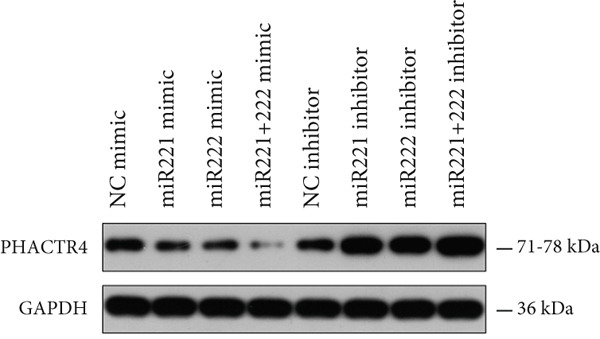
(c)

**Figure:**
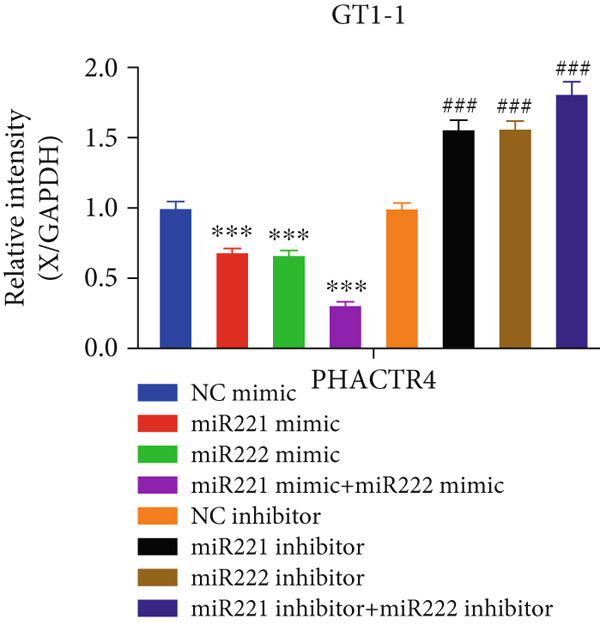
(d)

**Figure:**
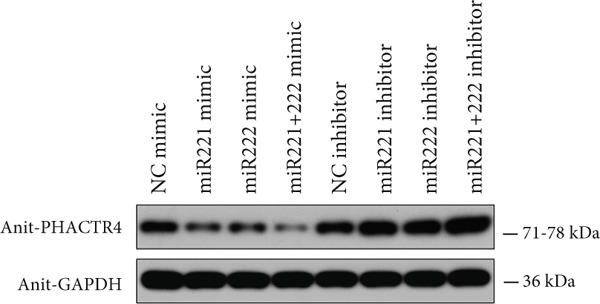
(e)

**Figure:**
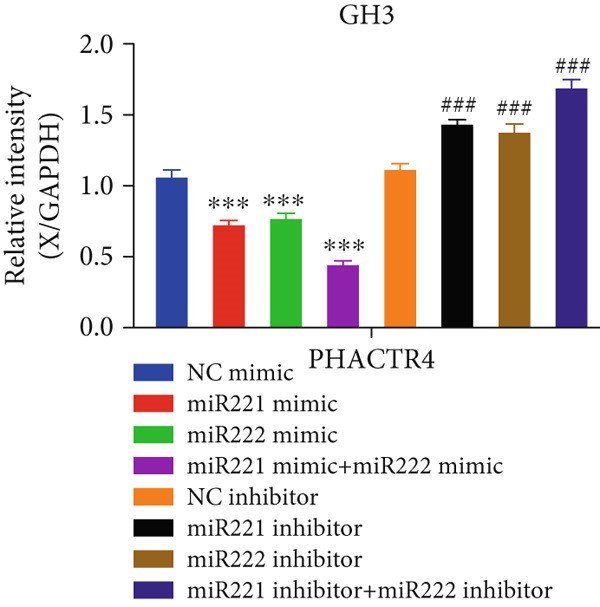
(f)

**Figure:**
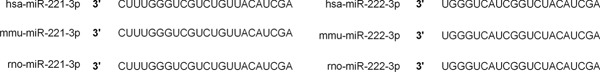
(g)

Luciferase reporter assay results revealed that fluorescence intensity of the wild‐type luciferase reporter plasmid was inhibited by transfection of miR‐221 mimic, miR‐222 mimic, and miR‐221/222 mimic while mutation of miRNA‐221/222 binding sites restrained this suppressive effect (*p* < 0.001), suggesting that miR‐221/222 could directly bind to the conserved sites in the 3 ^′^‐UTR regions of PHACTR4 mRNA, as shown in Figure 4b. Moreover, miRNA‐221/222 from human, mice, and rat demonstrate highly homology and the sequences are exactly identical (Figure 4g).

### 3.3. miRNA‐221/222 Promoted PA Progression In Vivo by Regulating PHACTR4

The effects of miR‐221/222 on PA growth in vivo were further explored and results revealed that tumor volumes and tumor weight were the highest at Day 28 in miR‐221/222 group compared with NC group, suggesting a synergistic effect of miR‐221 and miR‐222 in promoting PA progression in vivo (Figures 5a, 5b, 5c, 5d, 5e, and 5f). Western blot analysis validated miR‐221/222 overexpression downregulated PHACTR4, Cleaved‐Caspase3, E‐cadherin expression while upregulated N‐cadherin expression (Figures 5e, 5f, 5g, 5h, and 5i).

Figure 5microRNA‐221/222 promoted PA growth by targeting PHACTR4 in vivo. (a) Xenograft models were generated by injecting GH3 cells. At Day 28, all mice were sacrificed after anaesthetization and tumors were collected. (b) Tumor volume and body weight of nude mice in each group were measured every 3 days. (c–d) Tumor volume and tumor weight at Day 28 of the four groups. (e–i) microRNA‐221/222 inhibited PHACTR4, Cleaved‐Caspase3, and E‐cadherin and promoted N‐cadherin in nude mice. ∗ vs. miRNA‐NC *p* < 0.05; ∗∗ vs. miRNA‐NC *p* < 0.01; # vs. EmiR‐222 mimic *p* < 0.05; ## vs. miR‐222 mimic *p* < 0.01; ### vs. miR‐222 mimic *p* < 0.001.

**Figure:**
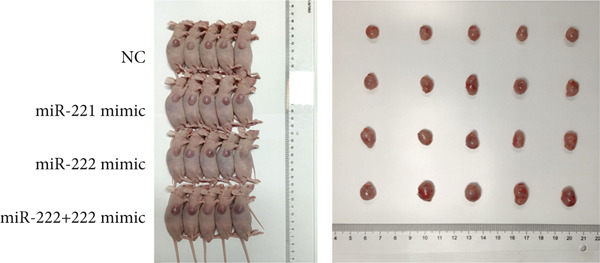
(a)

**Figure:**
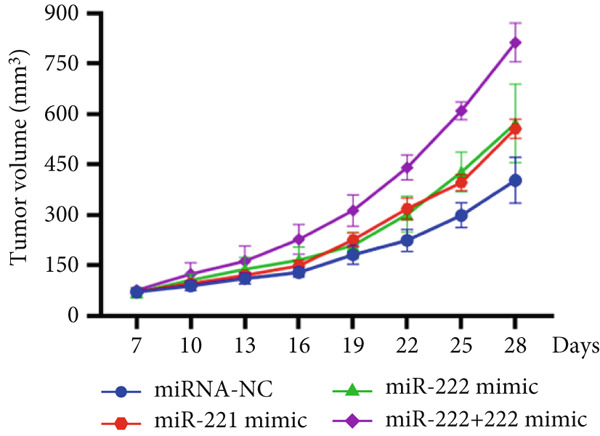
(b)

**Figure:**
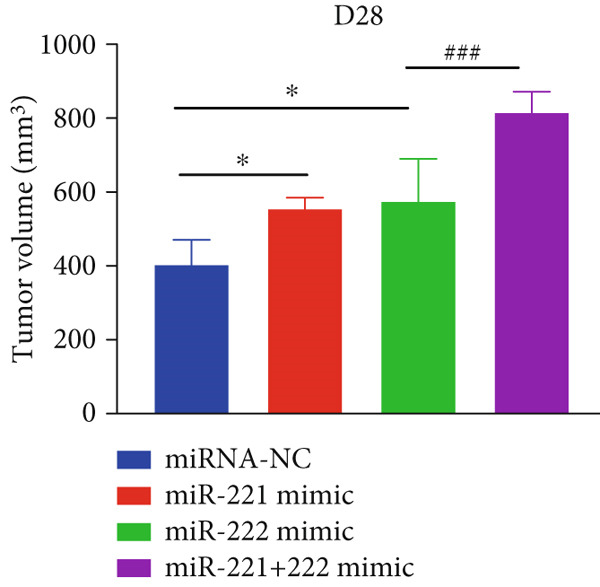
(c)

**Figure:**
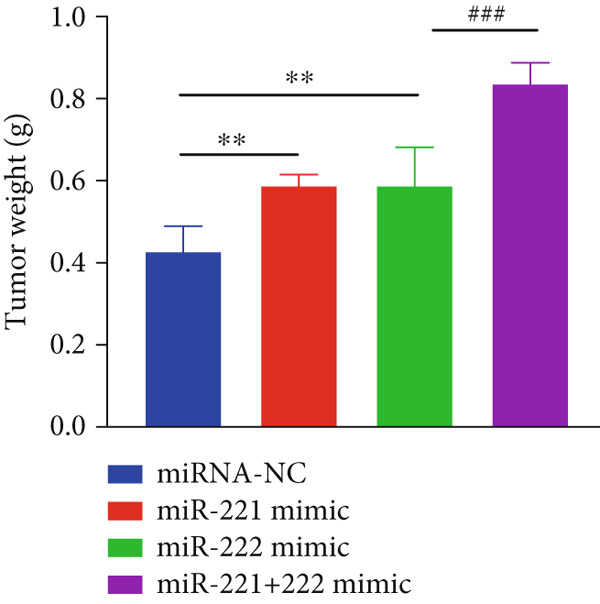
(d)

**Figure:**
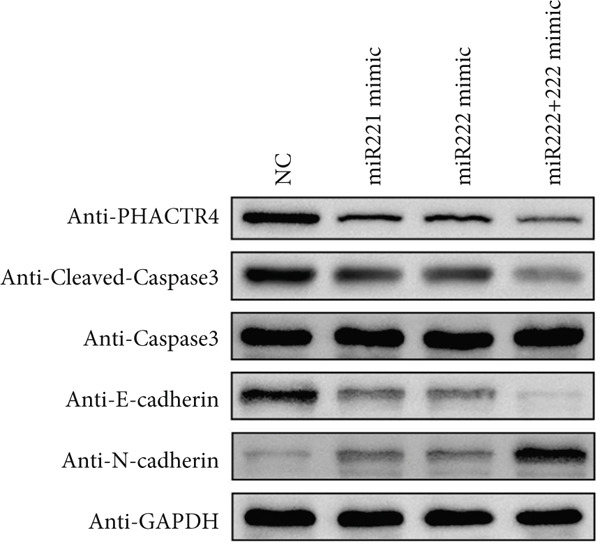
(e)

**Figure:**
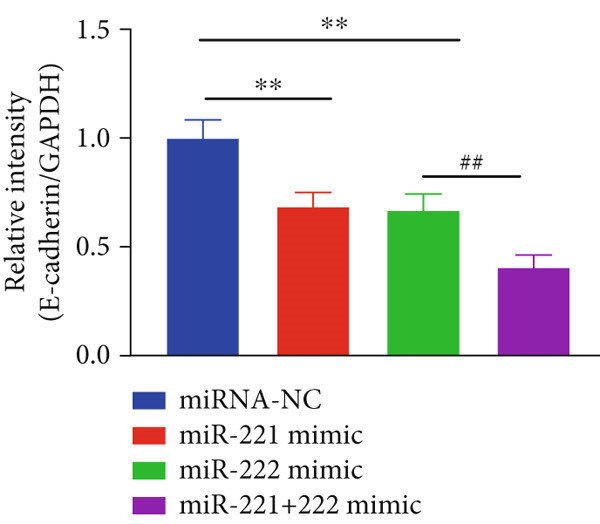
(f)

**Figure:**
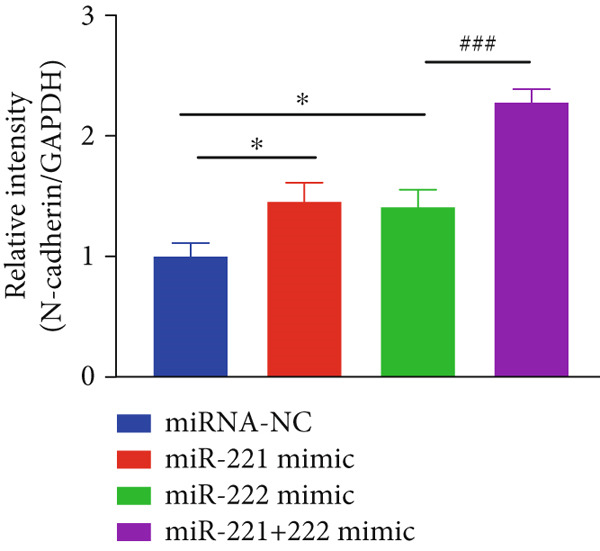
(g)

**Figure:**
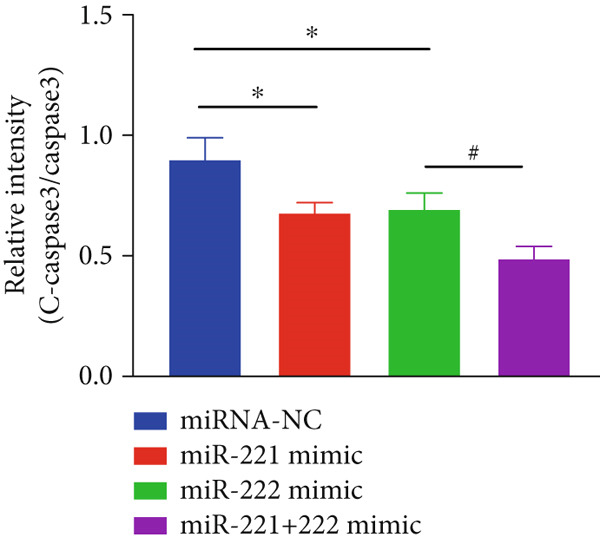
(h)

**Figure:**
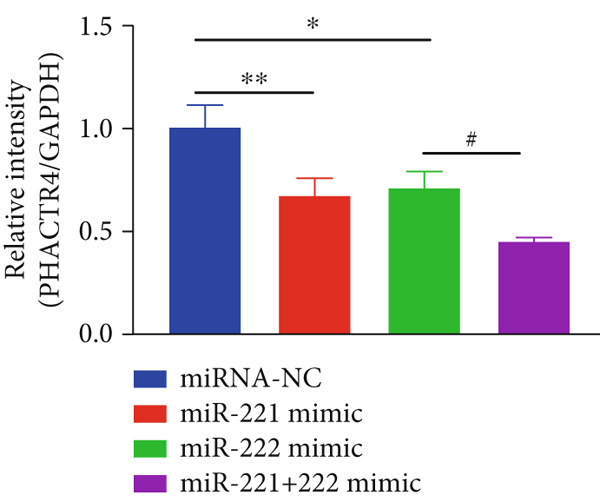
(i)

## 4. Discussion

miRNAs are noncoding single‐stranded RNA molecules, which do not participate in the transcription of genes themselves. Instead, they play a role in post‐transcriptional regulation by specifically binding to the 3 ^′^‐untranslated region of the target gene mRNA and degrading or blocking the translation of the target gene mRNA [19, 20]. Previous studies have found that miRNA‐30d and miRNA‐543 could act as proto‐oncogenes by inhibiting cell apoptosis and promoting cell invasion in PA, while miRNA‐149 and miRNA‐34a restrained cell proliferation, invasion, and accelerated cell apoptosis, playing the role of tumor suppressor genes [21–24]. Numerous studies have demonstrated the role of miR‐221/222 in human cancers and its potential clinical value as a promising therapeutic target [15, 25–28]. However, the role of miRNA‐221/222 in PA has not been discussed. In this study, we found that the expression level of miRNA‐221/222 in plasma exosomes from patients with pituitary tumor was higher than that from healthy people. Thus, we speculated that miRNA‐221/222 may be pro‐oncogene in PA progress.

Human miRNA‐221 and miRNA‐222 share a highly homologous core sequence in the X chromosome [25]. Given the high homology of miR‐221 and miR‐222 across species, including humans, mice, and rats, with identical mature and seed sequences, as shown in Figure 4g, GH3 (rat‐derived) and GT1‐1 (mouse‐derived) pituitary tumor cell lines were chosen to validate the functional effects of these miRNAs. The consistent biological outcomes observed in both models, including enhanced proliferation and migration and reduced apoptosis, highlight the evolutionary conservation of miR‐221/222 in pituitary tumor biology. This cross‐species validation strengthens the reliability of our findings and supports the translational relevance of targeting miR‐221/222 in human PAs.

Our results indicated that miRNA‐221/222 overexpression accelerated proliferation while suppressed apoptosis in PA cells, which was consistent with the outcomes of previous studies [15, 27]. In tumor, apoptosis is often inhibited, which makes abnormal or mutant cells unable to be eliminated, eventually leading to tumor formation [29]. Caspase‐3, the core member of Caspase family, is the key protein to perform cell apoptosis, playing an important role in the occurrence and development of many diseases such as tumor and ischemia‐reperfusion injury [30]. Cleaved‐Caspase‐3 is a cleavage fragment during the activation of Caspase‐3 protein, reflecting the activity of Caspase‐3 protein and level of apoptosis [31]. In our research, Western blot results showed that transfection of miRNA‐221/222 mimics suppressed expression levels of Cleaved‐Caspase3, partly unveiling the mechanism of suppressive effects of miRNA‐221/222 on apoptosis in PA cells.

Epithelial–mesenchymal transition (EMT) is a process in which epithelial cells gradually transform into mesenchyme‐like cells, playing an important role in tumor metastasis and invasion [32]. Low expression of epithelial marker such as E‐cadherin and high expression of mesenchymal marker such as N‐cadherin are associated with migration and invasion of a variety of tumors [33]. In our research, results indicated that overexpression of miRNA‐221/222 motivated migration, increased expression of N‐cadherin, and decreased expression of E‐cadherin, contributing to the role of proto‐oncogene of miRNA‐221/222 in PA. Unfortunately, in our research, both GT1‐1 and GH3 cells failed to invade across the membrane in Transwell invasion assay, showing no property of invasion. The effect of miRNA‐221/222 on invasion needs to be investigated in other PA cell lines in future research.

In order to elucidate the underlying mechanism of miRNA‐221/222 regulating tumor biology in PA cell lines, transcriptome sequencing was performed in four groups of GT1‐1 cell. GO functional enrichment analysis showed that the DEGs were mainly related to molecular function such as ubiquitin‐specific protease activity and biological process such as MAPK cascade activation, which were reported to regulate apoptosis, cell cycle, migration, and immune response [34–38]. KEGG enrichment pathway analysis demonstrated that DEGs were mainly enriched in Ras signaling pathway and regulation of actin cytoskeleton, which were crucial to cell proliferation, differentiation, migration, and invasion [39–43]. Therefore, our results provided new insights into the molecular mechanisms underlying the biological effects of PA and contributed to a better understanding of the development and progression of PAs. Next, Dataset GSE240781 from Gene Expression Omnibus on five patients with pituitary prolactinoma and three with normal pituitary tissue was used to verify and analyzing DEGs. Results showed that 18 genes in our DEGs were downregulated, all of which were potential target genes of miRNA‐221/222 in PA. Finally, through literature reviewing, we hypothesized that PHACTR4 was the target gene of miRNA‐221/222.

PHACTR4 overexpression plasmid was constructed and utilized to verify PHACTR4 was the target gene of miRNA‐221/222 in regulating tumor behavior of PA cell lines. Results indicated that PHACTR4 overexpression reversed the effects of miRNA‐221/222 on proliferation, apoptosis, and migration of PA cells. Meanwhile, dual luciferase reporter gene assay also confirmed that miRNA‐221/222 could directly bind to mRNA of PHACTR4. Moreover, xenograft experiment in nude mice demonstrated that the overexpression of PHACTR4 inhibited tumor growth in vivo. Thus, miRNA‐221/222 was proved to regulate proliferation, apoptosis, and migration of PA cells by targeting PHACTR4 in vivo and in vitro. As a tumor suppressor, PHACTR4 was reported significantly downregulated and mutated in many tumors, such as breast cancer, colorectal cancer, lung carcinoma, ovarian carcinoma, and renal cancer [17]. In our study, PHACTR4 was also found inhibited in the dataset GSE240781. In conclusion, miRNA‐221/222 could play a role as a proto‐oncogene in the development of pituitary tumors by targeting PHACTR4, providing a new target for the diagnosis and molecular treatment of pituitary tumors.

## Ethics Statement

This study was approved by the Ethics Committee of Hainan Hospital of Chinese PLA General Hospital (No. S2022‐420‐01). Written informed consent was obtained from each patient.

## Consent

The authors have nothing to report.

## Conflicts of Interest

The authors declare no conflicts of interest.

## Author Contributions

Weijun Gu, Wenjing Dong, and Shiju Yan conceived and designed the study, conducted the experiments, performed the statistical analyses, and prepared the manuscript. Wei Wang, Ping Pang, Lingyun Song, Di Sun, and Guoqing Yang contributed to the discussion section. Weijun Gu participated in the study’s design, supervised it, and provided commentaries on the manuscript text. All authors listed have made a substantial, direct, and intellectual contribution to the work and approved it for publication. Wenjing Dong, Shiju Yan, and Lingyun Song contributed equally to this work and share first authorship.

## Funding

This study was supported by Natural Science Foundation of Beijing, 7232155.

## Supporting Information

Additional supporting information can be found online in the Supporting Information section.

## Supporting information


**Supporting Information 1** Figure S1: microRNA‐221/222 promoted cell proliferation, migration, epithelial–mesenchymal transition, and inhibited apoptosis in GH3 cells.

**Supporting Information 2** Figure S2: The effects of PHACTR4 overexpression on the proliferation, apoptosis, and migration in GH3 cells transfected with microRNA‐221/222mimic.

**Supporting Information 3** Table S1: Sequences of synthesized microRNA.

## Data Availability

The data that support the findings of this study are available from the corresponding author upon reasonable request.
