# Supplementary figures and images for: miR‐221/222 Facilitate Pituitary Adenoma Progression Via PHACTR4 Downregulation
Source: Hum Mutat. 2026 Feb 5;2026:8584408. doi: 10.1155/humu/8584408 (PMC12876046; doi:10.1155/humu/8584408)

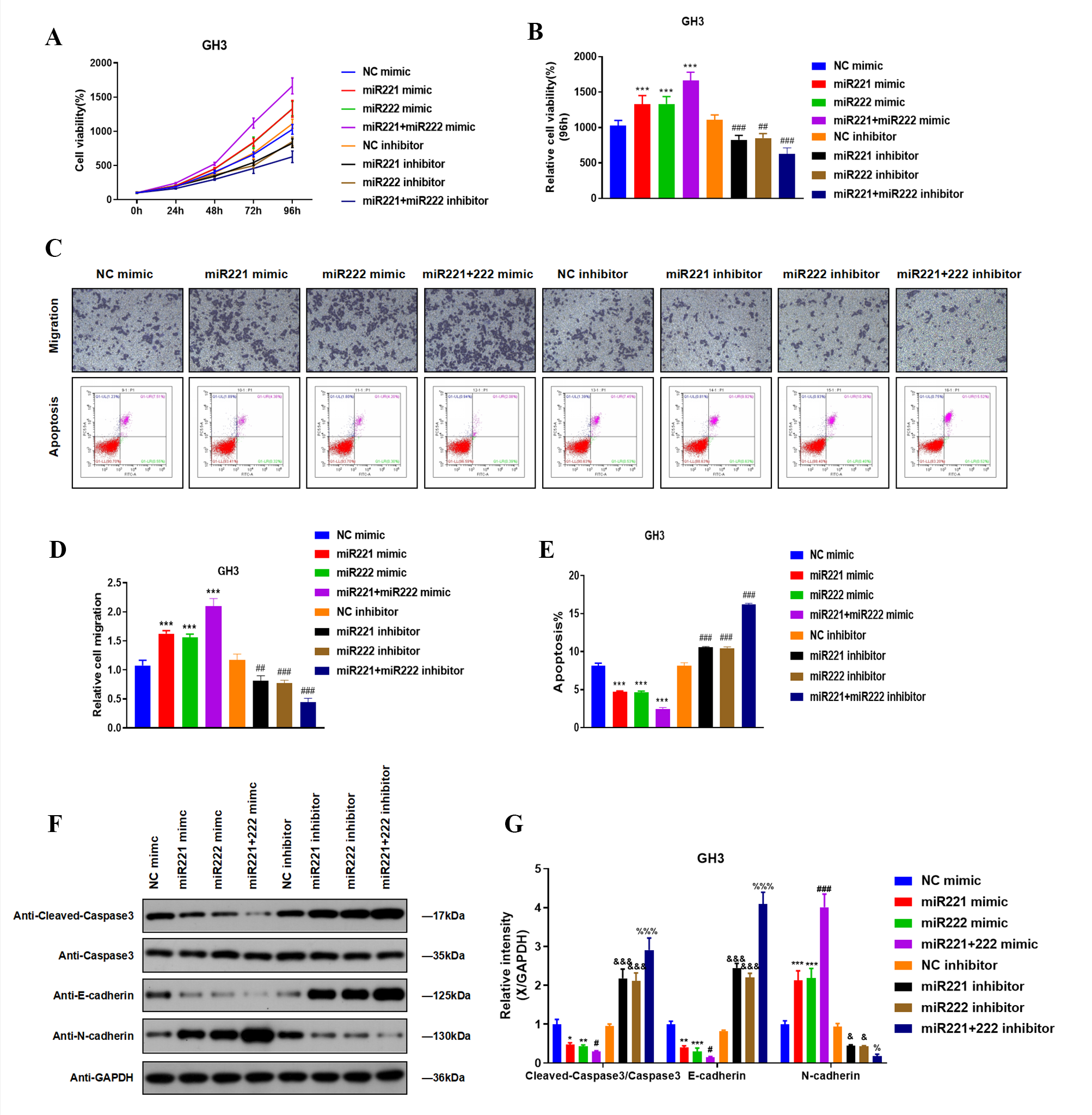

Supplement: Supplementary file 1 — Supporting Information 1 Figure S1: microRNA‐221/222 promoted cell proliferation, migration, epithelial–mesenchymal transition, and inhibited apoptosis in GH3 cells. [file HUMU-2026-8584408-s001.tif]

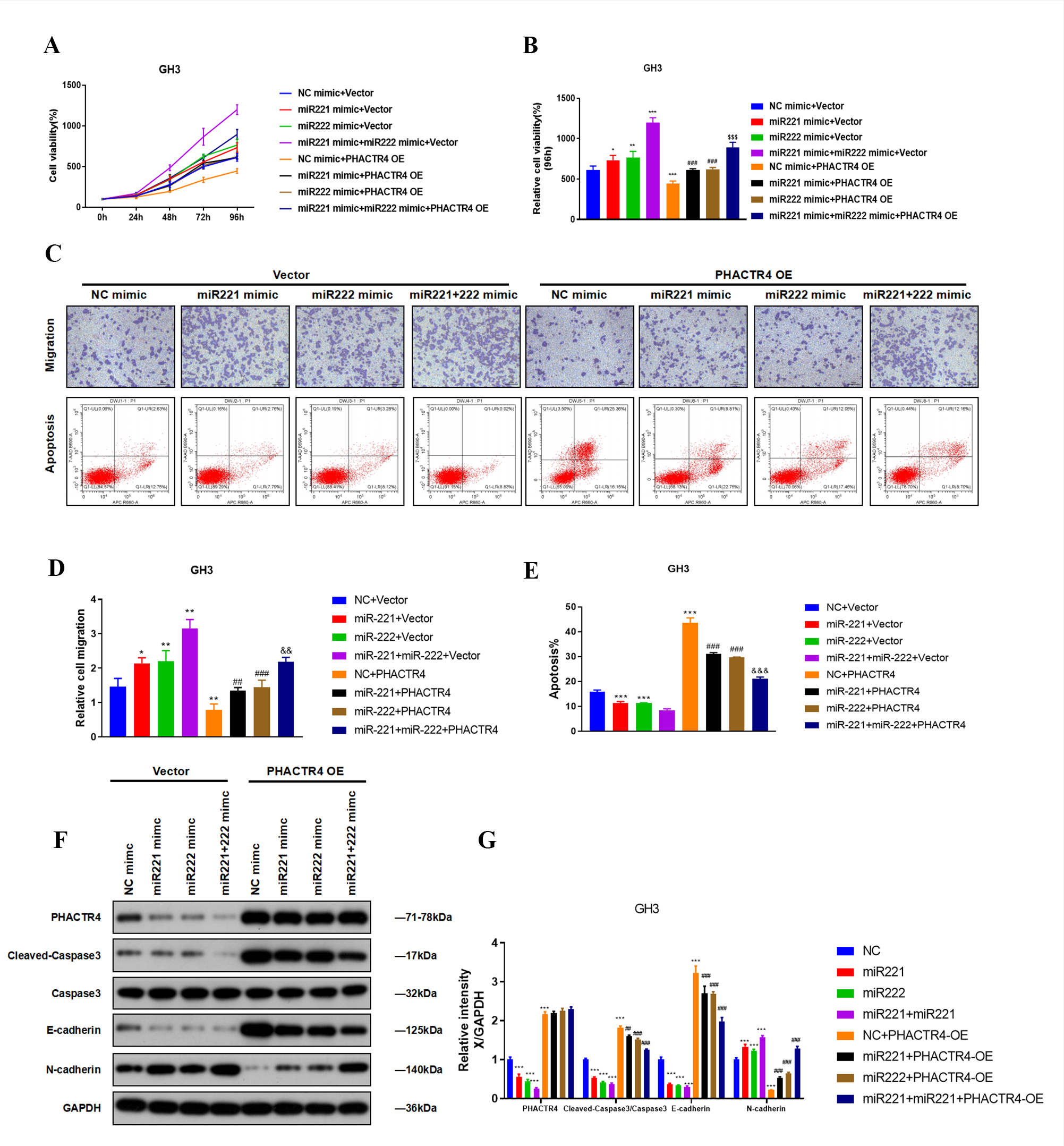

Supplement: Supplementary file 2 — Supporting Information 2 Figure S2: The effects of PHACTR4 overexpression on the proliferation, apoptosis, and migration in GH3 cells transfected with microRNA‐221/222mimic. [file HUMU-2026-8584408-s002.tif]
