# Supplementary material for: miR‐221/222 Facilitate Pituitary Adenoma Progression Via PHACTR4 Downregulation
Source: Hum Mutat. 2026 Feb 5;2026:8584408. doi: 10.1155/humu/8584408 (PMC12876046; doi:10.1155/humu/8584408)
Supplement: Supplementary file 3 — Supporting Information 3 Table S1: Sequences of synthesized microRNA. [file HUMU-2026-8584408-s003.docx]

**Table 1** Sequences of synthesized microRNA

| **microRNA** | **Sequences (5'-3')** |
| --- | --- |
| miR-221 mimic | AGCUACAUUGUCUGCUGGGUUUC |
| miR-221 inhibitor | GAAACCCAGCAGACAAUGUAGCU |
| miR-222 mimic | AGCUACAUCUGGCUACUGGGU |
| miR-222 inhibitor | ACCCAGUAGCCAGAUGUAGCU |
| mimic Negative Control | UCACAACCUCCUAGAAAGAGUAGA |
| inhibitor Negative Control | UCUACUCUUUCUAGGAGGUUGUGA |
